# Colonic Aging and Colorectal Cancer: An Unignorable Interplay and Its Translational Implications

**DOI:** 10.3390/biology14070805

**Published:** 2025-07-03

**Authors:** Qiyan Yin, Fen Qin, Fangliu Gan, Guangxi Zhao, Ronghua Chen, Yue Wen, Xueyang Hua, Fugui Zeng, Yuezheng Zhang, Yuliang Xiao, Wenbing Xie, Yong Tao

**Affiliations:** 1 Center for Life Sciences, Yunnan Key Laboratory of Cell Metabolism and Diseases, School of Life Sciences, Yunnan University, Kunming 650504, China; qiyanyin123@163.com (Q.Y.); fenqin0518@163.com (F.Q.); fangliugan123@163.com (F.G.); guangxizhao413@163.com (G.Z.); ronghuachen187@163.com (R.C.); fg_zeng@163.com (F.Z.); yuezhengzhang@gmail.com (Y.Z.); 2 Hefei National Laboratory for Physical Sciences at the Microscale, School of Basic Medical Sciences, Division of Life Sciences and Medicine, University of Science and Technology of China, Hefei 230026, China; yuewen123@mail.ustc.edu.cn (Y.W.); xyhua2000@gmail.com (X.H.); wxie6@ustc.edu.cn (W.X.); 3 Department of Gastroenterology, The Affiliated Hospital of Yunnan University, Kunming 650021, China; xiaoyuliang@ynu.edu.cn

**Keywords:** colon aging, colorectal cancer, biological aging, microenvironment, inflammation, dysbiosis, senescence, early detection, prevention, translational research

## Abstract

Colorectal cancer (CRC) incidence increases markedly with age, yet chronological age is an inadequate proxy for the complex biological processes involved. Colon aging, the intrinsic biological aging of the colonic tissue, is emerging as a crucial, active driver of CRC development. This review comprehensively analyzes the interplay between colon aging and CRC pathogenesis by examining fundamental hallmarks of aging—such as altered tissue homeostasis, epigenetic dysregulation, and microenvironmental shifts including chronic inflammation (inflammaging), gut microbiome dysbiosis, and extracellular matrix remodeling—manifest specifically within the aging colon to synergistically foster a pro-tumorigenic environment. Key findings synthesized from the literature highlight the critical roles of impaired colonic stem cell function, epithelial barrier disruption (“leaky gut”), persistent low-grade inflammation, and altered microbial communities and their metabolites in promoting CRC initiation and progression. Translating this mechanistic understanding holds significant promise: insights from colon aging research can inform novel biomarkers for improved early detection and risk stratification, guide the development of personalized preventative strategies and therapeutic interventions targeting aging pathways, and underpin public health initiatives focused on healthy colon aging. Ultimately, recognizing colon aging as a modifiable contributor to CRC offers a powerful avenue to potentially reduce CRC incidence and enhance patient outcomes, particularly in the vulnerable aging population.

## Simple Summary

The risk of colorectal cancer rises with age, but this is driven more by the biological aging of the colon than by chronological age alone. This review explores how the aging colon—with its weakened repair mechanisms, "leaky" protective barrier, chronic low-level inflammation, and disrupted microbial communities—creates an environment that fuels cancer development. This understanding of colon aging as a modifiable driver of cancer is profoundly important. It opens the door to new biomarkers for earlier detection, personalized prevention, and therapies that specifically target aging processes. Focusing on healthy colon aging is a powerful strategy to reduce cancer rates and improve outcomes for patients, especially older adults.

## 1. Introduction

Colorectal cancer (CRC) presents a major public health challenge, ranking as the second leading cause of cancer-related mortality in the US [1]. While CRC impacts all ages, it disproportionately affects older adults; approximately 90% of cases occur in individuals aged 50 and above [2]. Although overall incidence and mortality rates have declined, largely due to increased screening, the absolute number of cases in the aging population remains substantial, and disparities in access to care persist. Crucially, chronological age, while a strong statistical correlate, is an insufficient proxy for the complex biological processes driving cancer development [3,4]. A more nuanced understanding requires distinguishing between chronological age (the passage of time) and *biological age*—the cumulative molecular and cellular damage, functional decline, and altered tissue homeostasis that accrue over a lifespan [5,6].

In addition, significant gender differences exist in colorectal cancer (CRC) risk and the complex processes of colon aging, evident in various aspects [7]. Epidemiologically, males generally exhibit a higher overall CRC incidence, particularly for rectal and distal cancers, whereas older females may show a greater proportion of proximal colon cancers, and early-onset CRC also presents sex-specific trends [8]. These disparities are underpinned by a range of biological factors, including the distinct roles of sex hormones—where estrogens are often considered protective in females, while androgens’ impact in males is more complex—and the inherent differences in gut microbiome composition and function between sexes, influenced by hormones and lifestyle, which in turn affect inflammation and metabolism [9,10]. Furthermore, intrinsic variations in male and female immune systems contribute to differences in colonic inflammation (‘inflammaging’) and immune surveillance against tumors. Beyond internal biology, varying prevalences and impacts of metabolic and lifestyle factors like obesity, diabetes, smoking, and diet between genders also contribute to differential CRC risk [11]. Further emphasizing this complexity, recent multi-omics studies in non-human primates have begun to unravel the heterogeneity of intestinal aging by simultaneously considering age, sex, and proximal–distal anatomical variations, identifying specific molecular signatures and potential regulatory pathways like tryptophan metabolism that differ across these contexts [12]. While direct studies on sex differences in colonic cellular aging mechanisms (telomere shortening, senescence, DNA damage response) are less numerous, this remains an evolving area, with sex hormones and immune variations likely influencing these fundamental aging processes in a gender-specific manner [13].

Building upon this distinction, we introduce the concept of *colon aging*: the progressive accumulation of age-related biological changes *specifically within the colonic tissue*. This colon aging is driven by the hallmarks of aging, manifesting distinctly in the colonic environment as: altered tissue homeostasis, epigenetic dysregulation, genomic instability, gut microbiome dysbiosis, and chronic, low-grade inflammation (inflammaging) [14,15,16,17].

This review moves beyond simply acknowledging age as a risk factor. We explore the *active* and *dynamic* interplay between colon aging and CRC development. We will dissect how the hallmarks of aging, *within the colonic context*, create a pro-tumorigenic milieu. A central focus is the *translational significance* of this knowledge. By understanding the *mechanisms* by which age-related colonic changes increase CRC susceptibility, we can identify targets for innovative prevention, early detection, and treatment strategies, ultimately improving CRC management, particularly in the vulnerable older adult population [18]. We will synthesize current evidence on the molecular and cellular underpinnings of colon aging, its contribution to CRC pathogenesis, and potential avenues for intervention.

The remainder of this review is structured as follows: Section 2.1 details the characteristics and molecular mechanisms of colon aging, focusing on the hallmarks of aging within the colonic environment. Section 2.2 synthesizes the interactions between these age-related changes and CRC development. Section 2.3 explores the translational implications, discussing how understanding colon aging can inform improved CRC prevention, early detection, and treatment. Finally, Section 3 identifies future research directions, unresolved questions, and challenges/opportunities in this evolving field.

## 2. Defining Colon Aging: Beyond Chronological Time

**Figure 1:**
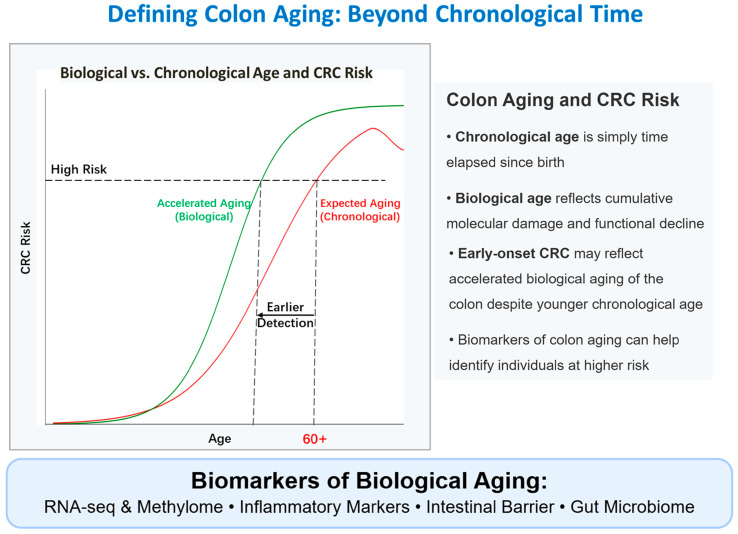
Defining colon aging: beyond chronological time. This figure illustrates the distinction between biological and chronological age in the context of colorectal cancer (CRC) risk. The graph (left panel) displays CRC Risk (Y-axis) plotted against Age (X-axis), comparing the trajectory for expected aging based on chronological age (red line) with that of accelerated biological aging (green line). It highlights that individuals undergoing accelerated biological aging reach a high CRC risk threshold at an earlier chronological age, potentially allowing for earlier detection. The text panel (right) explains that chronological age is simply time elapsed, whereas biological age reflects cumulative molecular damage and functional decline. It also notes that the colon may age at a different rate than other tissues and that accelerated biological aging might underlie early-onset CRC. Key categories of biomarkers used to assess biological aging, such as RNA-seq and methylome data, inflammatory markers, intestinal barrier integrity, and gut microbiome profiles, are listed at the bottom.

Building upon the distinction between chronological and biological age introduced earlier, it is crucial to consider how colon aging manifests uniquely. The colon’s dynamic nature, characterized by continuous epithelial renewal and constant interaction with a complex luminal environment, renders its biological aging trajectory particularly susceptible to modulation. This means the rate of biological aging within the colon can significantly diverge from an individual’s chronological age, a phenomenon visually conceptualized in Figure 1. This tissue-specific aging process is profoundly shaped by a host of factors, including genetic background, long-term dietary patterns, lifestyle choices, and environmental exposures, contributing to the heterogeneity observed in age-related CRC risk.

The potential dissociation between the colon’s biological state and calendar years holds particular relevance for understanding colorectal cancer (CRC) susceptibility (Figure 1). Emerging studies suggest that individuals diagnosed with early-onset CRC might display molecular characteristics indicative of accelerated biological aging within their colonic tissue, accumulating age-related damage faster than expected for their chronological age (Figure 1, left panel) [19]. Indeed, various biomarkers can reflect this biological aging process; alterations in DNA methylation patterns (‘epigenetic clocks’), specific gene expression signatures, chronic inflammatory markers, intestinal barrier function, and gut microbiome composition (Figure 1, bottom panel) are not only associated with normal colon aging but are often markedly dysregulated during CRC development [20]. This reinforces the principle that the *biological condition* of the colon, rather than merely the passage of time, is a critical determinant of vulnerability to CRC, potentially explaining variations in disease onset and risk [21] (Figure 1). A deeper understanding of these age-related biological changes within the colon is, therefore, paramount, beginning with an examination of the core hallmarks of aging in this specific context.

### 2.1. Hallmarks of Aging in the Colonic Context

#### 2.1.1. Altered Cell Fate and Tissue Homeostasis in the Aging Colon

The colonic epithelium is a dynamic tissue characterized by continuous self-renewal, driven by a population of intestinal stem cells (ISCs) residing at the base of crypts. These ISCs give rise to progenitor cells that differentiate into specialized cell types, including absorptive enterocytes, mucus-secreting goblet cells, and hormone-producing enteroendocrine cells, each with distinct functions essential for maintaining intestinal homeostasis [22,23]. However, this precisely orchestrated process of cell fate determination and differentiation becomes progressively dysregulated with age, leading to a decline in tissue function and an increased susceptibility to disease, including colorectal cancer [14,24]. A growing body of evidence points to intrinsic changes within the ISC compartment as a primary driver of age-related colonic dysfunction.

One of the most significant age-related alterations is a decline in ISC function. Studies in both mice and humans have shown that aging is associated with a reduced capacity for ISC self-renewal, impaired differentiation potential, and altered responses to niche signals [25,26,27]. This functional decline is often accompanied by transcriptional reprogramming, where aging ISCs exhibit altered expression of genes involved in cell cycle regulation, DNA repair, and metabolic processes [28,29,30]. Furthermore, research suggests that aging can lead to an increase in the number of ISCs, but these expanded stem cell pools often exhibit reduced functional capacity, suggesting a shift towards a more quiescent or dysregulated state [31]. This paradoxical increase in stem cell number, coupled with reduced function, highlights the complexity of age-related changes in the ISC compartment.

**Figure 2:**
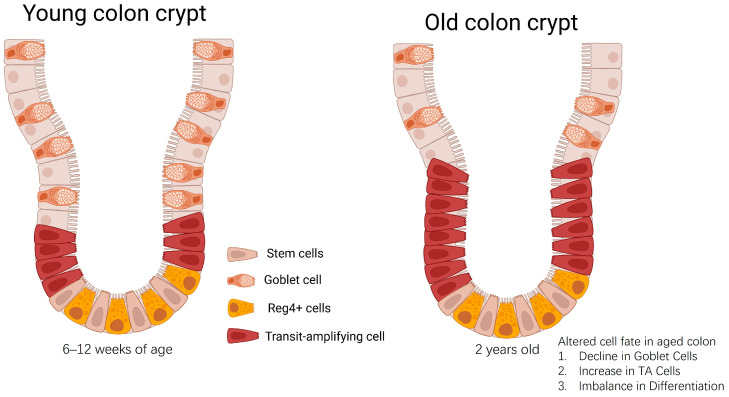
Age-related disruption of mouse colonic crypt homeostasis: altered cell fate. In the young mouse colonic crypt, stem cells and Reg4+ cells reside at the base, giving rise to proliferating transit-amplifying (TA) cells, which then differentiate into specialized cell types, including mucus-producing goblet cells, as they migrate up the crypt walls, maintaining tissue homeostasis. The aged mouse colonic crypt illustrates common age-related alterations, characterized by an expansion of the TA cell zone (indicated as “Increase in TA Cells”) and a reduction in the number of mature goblet cells (“Decline in Goblet Cells”). This represents an “Imbalance in Differentiation” and altered cell fate.

Beyond stem cell-intrinsic alterations, aging also profoundly impacts the differentiation process and lineage commitment of colonic epithelial cells. Studies have shown that aged ISCs exhibit altered differentiation trajectories, with a tendency towards skewed lineage output (Figure 2) [32,33]. This can manifest as a reduced proportion of mature enterocytes (responsible for nutrient absorption) or an imbalance in the ratio of secretory cell types, potentially disrupting the delicate balance of mucus production and barrier function [34,35]. This aberrant differentiation is not simply a passive consequence of stem cell aging; it is actively driven by changes in the expression of key transcription factors and epigenetic regulators that control cell fate decisions. The loss of transcriptional fidelity, characterized by a departure from the tightly controlled gene expression programs that define specific cell types, is increasingly recognized as a hallmark of aging, potentially due to the dysregulation of master regulators of cellular identity, including pioneer factors [36,37].

These alterations in cell fate—encompassing stem cell dysfunction, aberrant differentiation, and loss of cell identity—have profound consequences for tissue homeostasis in the aging colon. One critical consequence is the disruption of the epithelial barrier, often referred to as “leaky gut”. Age-related changes in cell junctions, reduced mucus production, and altered cell composition can compromise the integrity of the barrier, increasing permeability to luminal antigens, microbial products, and potential carcinogens [38,39]. This increased exposure, coupled with the chronic, low-grade inflammation (inflammaging) that characterizes the aging colon, creates a pro-tumorigenic microenvironment [40,41].

Finally, these changes in the epithelial compartment do not occur in isolation. Age-related changes influence all cell types, including immune cells, contributing to the overall aging microenvironment. Age-related alterations, including the changes highlighted above and dysregulation of other cell populations, lead to profound alterations in cell–cell communication in the colon. Crosstalk with stromal cells, immune cells, and the gut microbiome is also dysregulated with age, further contributing to tissue dysfunction. For example, senescent cells, which accumulate in the aging colon, secrete a complex mixture of factors known as the senescence-associated secretory phenotype (SASP), which can promote inflammation, disrupt tissue architecture, and influence the behavior of neighboring cells, including stem cells and immune cells [41,42,43,44]. The immune system itself undergoes significant age-related changes, contributing to chronic inflammation and altered surveillance.

#### 2.1.2. The Aging Colonic Microenvironment: A Foundation for Change

**Figure 3:**
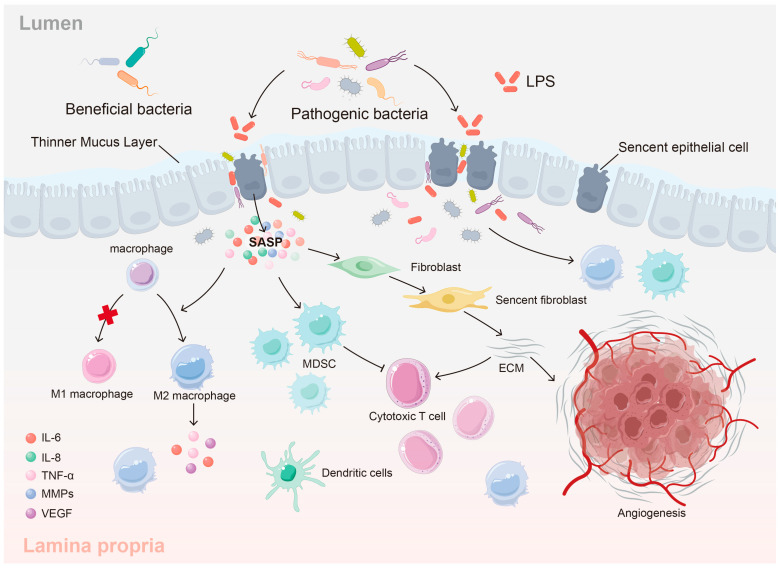
Interconnected factors in the aging colonic microenvironment promoting colorectal cancer development. This schematic illustrates the complex interplay of factors within the aging colonic microenvironment that collectively create a pro-tumorigenic ecosystem conducive to colorectal cancer (CRC) development. Key age-related changes depicted include: (1) Gut dysbiosis in the lumen, characterized by a decrease in beneficial bacteria and an increase in pathobionts. (2) Impaired epithelial barrier function (“leaky gut”) due to disrupted tight junctions, allowing translocation of bacterial products. (3) Accumulation of senescent cells (senescent fibroblasts) within the lamina propria that secrete a pro-inflammatory mix of factors known as the senescence-associated secretory phenotype (SASP). (4) A resulting chronic inflammatory environment involving various immune cells. (5) Immune dysregulation, featuring potentially increased M2 macrophages and myeloid-derived suppressor cells (MDSCs) that can suppress cytotoxic T-cell activity, leading to reduced anti-tumor immunity. (6) Remodeling of the extracellular matrix (ECM), including increased collagen deposition and stiffness, which alters cell–ECM interactions. (7) Increased angiogenesis (new blood vessel formation). These interconnected factors—dysbiosis, barrier dysfunction, senescence/SASP, inflammation, immune suppression, and ECM remodeling—contribute synergistically to CRC initiation and progression. Refer to the inset legend for definitions of specific symbols.

The aging colon is far more than simply a collection of aging epithelial cells; it is a dynamic and complex ecosystem. The colonic microenvironment, encompassing the extracellular matrix (ECM), resident immune cells, stromal cells (such as fibroblasts), the vasculature, and the vast community of the gut microbiome, undergoes profound and interconnected alterations with age. These changes, driven by the cellular and molecular processes described previously (cellular senescence, genomic/epigenetic instability, and altered cell fate), create a foundation for overall tissue dysfunction, setting the stage for age-related colonic diseases [45]. Key aspects of this transformed microenvironment include chronic inflammation (inflammaging), gut microbiome dysbiosis, ECM remodeling, and immune dysregulation—all intimately linked and mutually reinforcing (Figure 3).

A defining characteristic of the aging colonic microenvironment is “inflammaging,” a persistent, low-grade, sterile inflammatory state [41,46]. This chronic inflammation is not merely a passive consequence of the passage of time; it actively contributes to tissue damage and functional decline. Multiple, interconnected sources fuel colonic inflammaging. Senescent cells, which accumulate with age in both the epithelial and stromal compartments, are major contributors. These cells secrete a complex mixture of pro-inflammatory cytokines (such as IL-6 and IL-8), chemokines, growth factors, and proteases (including matrix metalloproteinases or MMPs)—collectively known as the senescence-associated secretory phenotype (SASP) (Figure 3) [47]. The SASP recruits and activates immune cells, creating a persistent inflammatory state, even in the absence of overt infection [48]. Concurrently, age-related disruption of intestinal barrier function, often described as “leaky gut,” contributes significantly. This increased permeability, resulting from alterations in cell junctions, reduced mucus production, and changes in epithelial cell composition, allows bacterial products (like lipopolysaccharide [LPS]) and other luminal antigens to translocate into the colonic mucosa [49]. This constant exposure activates pattern recognition receptors (PRRs), including Toll-like receptors (TLRs), on both epithelial and immune cells, further amplifying the inflammatory cascade and creating a positive feedback loop (Figure 3) [50,51].

The gut microbiome, the vast and diverse community of microorganisms residing within the colon, also undergoes dramatic shifts with age, a condition referred to as dysbiosis. This age-related dysbiosis is typically characterized by a decrease in microbial diversity, a reduction in the abundance of beneficial bacteria (such as *Bifidobacterium* and *Lactobacillus* species—key producers of the anti-inflammatory short-chain fatty acid butyrate), and a concurrent increase in potentially pathogenic or opportunistic bacteria (pathobionts) [52,53,54]. These microbial shifts are not simply a consequence of aging; they actively modulate the altered colonic environment. Dysbiotic bacteria are observed to exacerbate inflammaging by directly producing pro-inflammatory metabolites and activating PRRs [55,56]. Certain bacterial species can degrade the protective mucus layer and disrupt tight junctions, further compromising barrier integrity and increasing exposure to luminal contents [57,58]. The microbiome further impacts functions such as bile acid metabolism and fucosylation, adding further complexity to the changes [59,60]. This complex interplay is bidirectional: age-related physiological changes (decreased motility, altered immune responses, and dietary changes) influence the microbiome’s composition and function, while the altered microbiome, in turn, contributes to age-related changes in the colon.

The extracellular matrix (ECM) of the colon, far from being a mere inert structural scaffold, is a dynamic regulator of cellular behavior. With aging, the colonic ECM undergoes extensive remodeling, characterized by changes in its composition, organization, and mechanical properties [61,62,63]. A hallmark of this remodeling is increased collagen deposition and cross-linking, often driven by the activity of senescent fibroblasts and their associated SASP [41,64,65]. This leads to tissue fibrosis. This altered ECM is not simply structural. The aging colon also exhibits altered levels and distribution of other ECM components, including proteoglycans, glycoproteins (such as fibronectin and laminin), and matricellular proteins [61,66]. Furthermore, the activity of matrix metalloproteinases (MMPs), enzymes responsible for ECM degradation and remodeling, is often dysregulated in the aging colon, with some MMPs upregulated and others downregulated (Figure 3) [67,68]. These alterations impact tissue architecture, mechanical properties, and signaling, creating an environment which alters cell–cell and cell–matrix interactions.

The aging colonic microenvironment is also characterized by significant immune dysregulation, encompassing both “immune senescence” (a decline in adaptive immunity) and the aforementioned “inflammaging” (an increase in innate immune activation and chronic inflammation) [46,52]. An age-related decline in the number and function of T-lymphocytes and B-lymphocytes, key components of the adaptive immune system, impairs the ability to clear pathogens [69,70]. Paradoxically, aging is also associated with increased activation of innate immune cells, such as macrophages and neutrophils, further contributing to chronic, low-grade inflammation [46,71]. The aging colon often exhibits an altered cytokine balance, shifting towards a pro-inflammatory profile. Furthermore, there are changes to specific immune cells, such as increased levels of MHC-II [72]. The interplay between different immune cells is altered, and the communication between cells becomes dysregulated [73,74]. These combined alterations lead to a dysregulated immune system in the colon, contributing to reduced tissue health.

#### 2.1.3. Epigenetic Dysregulation and Genomic Instability in the Aging Colon

The aging colon is characterized by increasing genomic instability, a state of elevated susceptibility to genetic alterations. This instability arises from a combination of increased DNA damage and decreased DNA repair capacity. As colonic epithelial cells age, they accumulate DNA damage from both endogenous sources (reactive oxygen species produced during metabolism, errors during DNA replication) and exogenous sources (dietary carcinogens, radiation) [75]. Simultaneously, the efficiency of DNA repair pathways, including base excision repair, nucleotide excision repair, and mismatch repair, declines with age. This combination leads to an accumulation of mutations, deletions, insertions, and chromosomal aberrations. Telomere shortening, another hallmark of aging, also contributes to chromosomal instability in the aging colon. The resulting genomic instability creates a “mutator phenotype,” increasing the likelihood of acquiring the driver mutations that initiate and promote CRC development.

The aging process is also inextricably linked to profound alterations in the epigenome, encompassing changes in DNA methylation, histone modifications, and chromatin architecture [14,76,77]. These epigenetic modifications, unlike genetic mutations, do not alter the DNA sequence itself, but rather influence how genes are expressed. In the context of the aging colon, these alterations are not merely passive bystanders; they act as master regulators, orchestrating and integrating the effects of multiple other hallmarks of aging, ultimately contributing to cellular dysfunction, loss of tissue homeostasis, and increased cancer susceptibility [78,79].

One of the most extensively studied epigenetic modifications is DNA methylation, the addition of a methyl group to a cytosine base, typically at CpG dinucleotides. The aging colon exhibits a complex pattern of DNA methylation changes, often described as “epigenetic drift” [80,81]. This includes a global loss of DNA methylation (hypomethylation), particularly in repetitive regions of the genome, which can contribute to genomic instability [82]. Paradoxically, aging is also associated with increased DNA methylation (hypermethylation) at specific gene promoters, often within CpG islands [76,83,84]. These CpG islands are frequently located near the start sites of genes, and their hypermethylation can lead to transcriptional silencing of key genes involved in tumor suppression, DNA repair, cell cycle control, and differentiation [85,86]. Importantly, some of these age-related DNA methylation changes are observed in normal-appearing colonic mucosa before the development of any visible lesions, suggesting that they represent early events in the aging process that predispose to cancer [87,88]. Furthermore, studies have shown that the rate of epigenetic aging, as measured by DNA methylation “clocks,” can differ between individuals and even between different regions of the colon (proximal vs. distal), potentially contributing to the heterogeneity of CRC risk [76,89,90].

Beyond DNA methylation, age-related alterations in histone modifications also play a critical role in colon aging. Histones are proteins around which DNA is wrapped to form chromatin, and their modifications (acetylation, methylation, phosphorylation) can influence chromatin structure and gene accessibility [91]. Aging is associated with both global changes in histone modification patterns (loss of certain histone marks) and specific changes in individual genes [92,93]. These alterations can disrupt the balance between euchromatin (open, transcriptionally active) and heterochromatin (condensed, transcriptionally silent), leading to aberrant gene expression [79]. For example, decreased levels of histone acetylation (a mark of active transcription) at the promoters of tumor suppressor genes have been observed in the aging colon, potentially contributing to their silencing [94]. Conversely, increased levels of certain histone methylation marks (H3K27me3, associated with gene repression) have been linked to the silencing of genes involved in differentiation and tissue homeostasis [95]. The interplay between different histone modifications, and between histone modifications and DNA methylation, adds further complexity to the epigenetic landscape of the aging colon.

In essence, epigenetic dysregulation acts as a central hub, receiving input from various age-related stresses (oxidative stress, DNA damage, inflammation) and translating these signals into altered gene expression patterns that drive cellular dysfunction and contribute to the pro-tumorigenic microenvironment of the aging colon. Understanding the specific epigenetic alterations that occur in the aging colon, and how they interact with other aging hallmarks, is crucial for developing effective strategies to prevent and treat CRC.

### 2.2. Interactions Between Colon Aging and Colorectal Cancer Development

#### 2.2.1. Consequence of Cell Fate and Homeostasis Disruption: Link to Tumorigenesis

**Figure 4:**
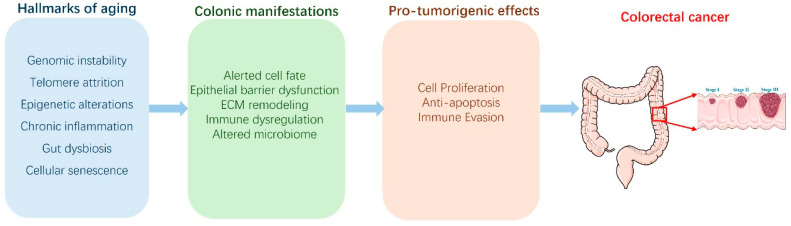
Molecular and cellular pathways linking colon aging hallmarks to colorectal cancer development. This diagram illustrates the proposed molecular and cellular pathways connecting general hallmarks of aging (left column) to the initiation and progression of colorectal cancer (CRC, far right). Key aging hallmarks, including genomic instability, telomere attrition, epigenetic alterations, cellular senescence, chronic inflammation, and gut dysbiosis, lead to specific colonic manifestations (middle column) such as altered cell fate, epithelial barrier dysfunction (“leaky gut”), extracellular matrix (ECM) remodeling, immune dysregulation, and an altered microbiome. These age-related changes within the colon subsequently promote pro-tumorigenic effects (right column), including increased cell proliferation, reduced apoptosis, genomic instability, and immune evasion. Together, these effects contribute to the development of CRC.

The age-related alterations in colonic cell fate and tissue homeostasis, described in the previous section, are not simply passive consequences of aging; they actively create a microenvironment that is highly permissive for the initiation and progression of colorectal cancer (CRC) [96,97]. The disruption of normal cellular processes, driven by the interconnected hallmarks of aging, establishes a cascade of events that ultimately increase the likelihood of malignant transformation (Figure 4).

One of the most direct consequences of altered cell fate is the disruption of the normal balance between cell proliferation, differentiation, and apoptosis. Dysfunctional stem cells, with impaired self-renewal and differentiation capacity, may give rise to a population of cells that are less differentiated, more proliferative, and more resistant to programmed cell death [98]. This shift in cellular dynamics creates a pool of cells that are more susceptible to acquiring further genetic and epigenetic alterations. Furthermore, the loss of cell identity and transcriptional fidelity, potentially driven by the dysregulation of key transcription factors, can lead to the aberrant expression of genes that promote uncontrolled growth and survival [99,100]. The differentiated cells are also impacted by the loss of cell fate control.

The breakdown of tissue homeostasis, particularly the disruption of the epithelial barrier (“leaky gut”), further exacerbates these effects. Increased intestinal permeability allows luminal contents, including bacteria, bacterial products (lipopolysaccharide [LPS]), and dietary carcinogens, to penetrate the colonic mucosa and interact directly with epithelial cells and underlying immune cells [39,49]. This chronic exposure to pro-inflammatory and genotoxic stimuli can accelerate the accumulation of DNA damage and epigenetic alterations, driving the transformation of already vulnerable cells. The altered signaling, such as Wnt signaling alterations, also contributes [101].

In summary, the age-related disruption of cell fate and tissue homeostasis in the colon creates a “perfect storm” for CRC development. The combination of increased cellular vulnerability (due to stem cell dysfunction, aberrant differentiation, and loss of cell identity) provides fertile ground for the initiation and progression of colorectal cancer.

#### 2.2.2. The Aging Colonic Microenvironment: A Crucible for CRC Development

The aging colon is far more than simply a passive collection of aging epithelial cells; it’s a dynamically changing ecosystem, a complex microenvironment where a confluence of factors creates conditions highly conducive to colorectal cancer (CRC) initiation and progression [97]. While the cellular and molecular changes within epithelial cells (discussed previously) are critical, it is within the broader microenvironment—encompassing the extracellular matrix (ECM), immune cells, stromal cells like fibroblasts, the vasculature, and the gut microbiome—that these age-related alterations exert their most potent pro-tumorigenic effects (Figure 4). These microenvironmental components are not independent entities; they exist in a state of constant, reciprocal interaction, a dynamic network that collectively drives age-related tissue dysfunction and elevates CRC risk.

As mentioned above, a key feature of the aging colonic microenvironment is chronic inflammation. The consequences of this chronic inflammation are far-reaching and profoundly pro-tumorigenic, directly promoting CRC by inducing DNA damage (via reactive oxygen and nitrogen species), stimulating cell proliferation, inhibiting apoptosis, fostering angiogenesis, and upregulating oncogenes [102,103,104]. Inflammatory signalling also affects metabolic reprogramming [104].

In addition, dysbiosis of the intestinal flora that occurs during aging has been linked to the development of CRC. For example, some gut bacteria can metabolize dietary components into carcinogenic compounds, directly increasing DNA damage and mutation risk in colonic epithelial cells. The microbiome is also related to altered bile acid metabolism and altered epithelial cell fucosylation. This complex interplay is bidirectional: age-related physiological changes (reduced intestinal motility, altered immune function, dietary changes) influence the microbiome’s composition and function, while the altered microbiome, in turn, exacerbates age-related changes and promotes CRC.

Building upon the understanding that the extracellular matrix (ECM) of the colon undergoes substantial age-related remodeling [105,106], this altered ECM is not simply a structural byproduct of aging; it actively influences cell signaling. A stiffer ECM can activate signaling pathways (such as integrin signaling and YAP/TAZ signaling) that promote cell proliferation, survival, migration, and invasion—all hallmarks of cancer [107,108]. Furthermore, changes in ECM composition disrupt normal cell–cell and cell–matrix interactions, contributing to the loss of tissue homeostasis. The ECM also plays a critical role in regulating immune cell infiltration and function, acting as both a physical barrier and a signaling hub, with a stiffer ECM potentially impairing immune cell infiltration and promoting the accumulation of immunosuppressive cells [109].

Finally, profound dysregulation of immune function is one of the key features of the aging colonic microenvironment. Age-related decline in the number and function of cytotoxic T-lymphocytes (CTLs) and natural killer (NK) cells impairs the immune system’s ability to effectively recognize and eliminate pre-cancerous or cancerous cells [110], leading to compromised immune surveillance. Simultaneously, the chronic, low-grade inflammation, fueled by senescent cells, dysbiosis, and leaky gut, creates a pro-tumorigenic milieu. The aging colon often exhibits an increased infiltration of myeloid-derived suppressor cells (MDSCs) and tumor-associated macrophages (TAMs), particularly M2-polarized macrophages, which actively suppress anti-tumor immune responses and promote tumor growth, angiogenesis, and metastasis through various mechanisms, including the secretion of immunosuppressive cytokines (IL-10, TGF-β) and the expression of immune checkpoint molecules (PD-L1) [111]. Furthermore, aging impacts the crucial communication and interactions between all these cells.

In summary, the aging colonic microenvironment represents a complex and dynamic interplay of chronic inflammation, gut microbiome dysbiosis, ECM remodeling, and immune dysregulation. These interconnected factors create a “perfect storm,” a highly permissive milieu that promotes the initiation, progression, and metastasis of CRC. Understanding the specific mechanisms by which these age-related microenvironmental changes interact is crucial for developing effective strategies for CRC prevention and treatment, particularly in the growing elderly population.

#### 2.2.3. Genomic and Epigenetic Instability as Drivers of Transformation

The accumulated DNA damage and epigenetic dysregulation characteristic of the aging colon create a state of genomic and epigenomic instability that directly drives the transformation of normal colonic epithelial cells into pre-cancerous and, ultimately, cancerous cells [112,113,114,115]. This instability acts as a powerful engine for tumorigenesis, increasing the likelihood of acquiring the critical genetic and epigenetic alterations that define the malignant phenotype.

One of the primary consequences of genomic instability is an elevated mutation rate. As discussed previously, aging is associated with both increased DNA damage and impaired DNA repair capacity [116,117,118]. This combination creates a “mutator phenotype,” where cells accumulate mutations at a faster rate than normal [119]. While many of these mutations may be inconsequential, some will inevitably occur in genes that control cell growth, proliferation, and apoptosis. These “driver” mutations confer a selective advantage to the affected cells, allowing them to outcompete their normal neighbors and form clonal expansions [120,121]. In the context of colorectal cancer, mutations in key tumor suppressor genes (APC, TP53, SMAD4) and oncogenes (KRAS, BRAF) are frequently observed, and their acquisition often follows a characteristic sequence during the progression from normal epithelium to adenoma to carcinoma (the “adenoma–carcinoma sequence”) [122,123]. The aging colon, with its heightened genomic instability, provides a fertile ground for the accumulation of these driver mutations [121].

Epigenetic alterations, particularly DNA methylation changes, play an equally critical, and often earlier, role in driving transformation [124]. As detailed in Section 2.1.3, the aging colon exhibits widespread changes in DNA methylation, including global hypomethylation and focal hypermethylation at CpG islands within gene promoters [125]. This aberrant DNA methylation can silence tumor suppressor genes, even in the absence of mutations, effectively mimicking the effect of a genetic loss-of-function mutation [126,127]. Furthermore, DNA methylation changes can alter the expression of genes involved in DNA repair, cell cycle control, and differentiation, further contributing to genomic instability and altered cell fate [128]. Epigenetic silencing can, therefore, function as an initiating event, predisposing cells to further genetic and epigenetic alterations.

Importantly, genomic and epigenomic instability are not independent processes; they are intricately intertwined. DNA damage can directly influence the epigenome, and epigenetic alterations can, in turn, affect DNA repair and genomic stability [129,130]. For example, oxidative DNA damage can recruit DNA methyltransferases, leading to aberrant methylation at the site of damage [131]. Conversely, epigenetic silencing of DNA repair genes can increase the cell’s susceptibility to DNA damage and mutation. This interplay creates a vicious cycle, where genomic and epigenetic instability mutually reinforce each other, accelerating the accumulation of alterations that drive tumorigenesis. The combination of genetic mutations and epigenetic silencing is a potent force in the transformation of normal colonic epithelial cells into cancer cells [132,133]. A thorough understanding of these intricate molecular and cellular mechanisms by which colon aging contributes to CRC is, therefore, not only fundamentally important but also provides a critical foundation for developing novel translational strategies to combat this disease.

### 2.3. Translational Significance of Colon Aging and Colorectal Cancer Research

The detailed mechanistic insights into how colon aging actively drives colorectal cancer pathogenesis, as discussed in the preceding sections, carry profound translational implications. Harnessing this knowledge offers promising avenues to enhance CRC prevention, improve the accuracy of early detection and risk assessment, and develop novel therapeutic interventions, particularly for the vulnerable aging population. The following sections will explore these translational opportunities in greater detail.

#### 2.3.1. Improving Early Detection and Risk Stratification for Colorectal Cancer

**Figure 5:**
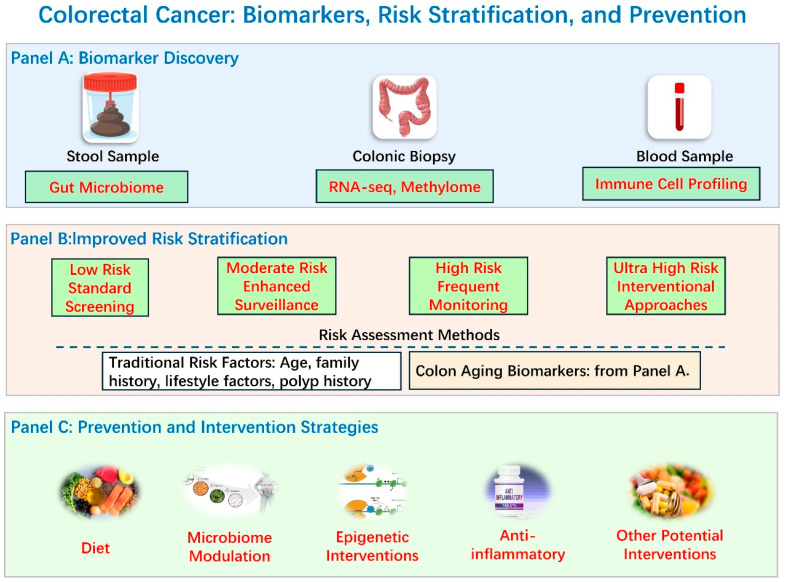
Translating colon aging insights into colorectal cancer prevention, early detection, and risk stratification. This figure outlines translational applications derived from understanding the interplay between colon aging and colorectal cancer (CRC). Panel A: Biomarker discovery illustrates potential sources and types of colon aging-related biomarkers relevant to CRC risk and detection. These include analyzing the gut microbiome from stool samples, assessing transcriptomic (RNA-seq) and epigenomic (methylome) profiles from colonic biopsies, and performing immune cell profiling from blood samples. Panel B: Improved risk stratification demonstrates how incorporating these novel colon aging biomarkers (from Panel A) with traditional risk factors (such as age, family history, lifestyle, and polyp history) can enhance CRC risk assessment. This improved stratification allows for categorizing individuals into different risk levels (low, moderate, high, ultra-high), guiding personalized management strategies that range from standard screening and enhanced surveillance to frequent monitoring or specific interventional approaches. Panel C: Prevention and intervention strategies highlights potential approaches informed by colon aging research aimed at preventing CRC or intervening in high-risk individuals. These strategies include dietary modifications, microbiome modulation (prebiotics, probiotics, fecal microbiota transplantation), epigenetic interventions, the use of anti-inflammatory therapies, and other potential interventions targeting aging pathways.

Advancements in understanding the aging colon are poised to revolutionize colorectal cancer (CRC) early detection and risk stratification, benefiting individuals of all ages, but with particular significance for older adults who bear the highest burden of this disease (Figure 5). These advancements span the development of novel biomarkers, refined risk prediction models, optimized screening strategies, and non-invasive detection technologies. Research into the aging colon is uncovering a wealth of potential biomarkers that could significantly improve early CRC detection across the entire population. Age-related changes in DNA methylation patterns [134], such as those observed in the *SDC2* gene, hold promise for non-invasive stool-based testing [135]. Similarly, circulating cell-free nucleosomes (ccfn) carrying epigenetic modifications show potential, although combining them with other factors like CEA, age, and sex may be necessary for optimal performance [136]. Circulating tumor DNA (ctDNA) analysis, particularly focusing on methylation haplotype patterns (ColonES), demonstrates high sensitivity and specificity for detecting both advanced adenomas and CRC [137]. Identification of age-related differentially expressed genes, such as *DLX2* and *PCOLCE2*, offers additional avenues for biomarker development [138]. The aberrant expression of miRNAs in tumor tissue and potentially plasma shows promise in diagnosis and prognosis, even for stage II and III CRC [139]. Furthermore, shifts in the gut microbiome composition and function are associated with both aging and CRC. Identifying specific microbial signatures, such as increased levels of p-cresol and 3(4H)-DBZ in fecal samples [140,141], or patterns of microbial gene expression [142], could provide non-invasive markers for early detection. Investigation into age-related changes in colonic cells, beyond molecular aspects, including epithelial cells and fibroblasts, may identify cellular phenotypes indicative of increased CRC risk.

A deeper understanding of colon aging can also lead to more accurate CRC risk prediction models applicable across the lifespan. Incorporating biomarkers of colon aging into existing risk assessment tools could identify individuals with “accelerated” colon aging, who may benefit from earlier or more frequent screening, regardless of their chronological age. This personalized approach would allow for more targeted prevention and screening strategies. This research can also inform the development of age-adjusted or risk-adapted screening protocols that are more effective and cost-efficient. For instance, studies suggest that extending the upper age limit for screening may be more cost-effective than lowering the starting age, provided there is sufficient colonoscopy capacity [143]. Risk stratification based on factors like fecal hemoglobin concentration in FIT testing could allow for personalized screening intervals [144], potentially reducing unnecessary colonoscopies, and while national guidelines exist, optimal screening age and frequency may vary [145], highlighting regional adaption.

The development of liquid biopsies (blood-based ctDNA tests [146] and improved stool-based tests [147]) offers particularly significant advantages, especially for older adults who may be less tolerant of invasive procedures like colonoscopy. These non-invasive methods can increase screening participation and improve early detection rates in this high-risk population. Older adults will disproportionately benefit from these advancements. They are at the highest risk of developing CRC, and current screening can be challenging due to comorbidities, reduced tolerance for invasive procedures, and concerns about over-screening in individuals with limited life expectancy. Improved risk stratification, optimized screening protocols, and non-invasive detection technologies will enable more personalized and effective CRC prevention and early detection strategies for this vulnerable population [148,149].

#### 2.3.2. Developing Preventative Interventions and Promoting Healthy Colon Aging

Research into the aging colon is not only illuminating the mechanisms of disease but also opening avenues for prevention and the promotion of healthy colon aging for all, with particular relevance to the older population, who experience the highest risk of colorectal cancer (CRC). This involves a multi-pronged approach encompassing lifestyle and dietary modifications, potential pharmacological interventions, and informed public health strategies. A cornerstone of colon health, regardless of age, is a healthy lifestyle and diet. Research consistently demonstrates the protective effects of increased dietary fiber, particularly from fruits, vegetables, and whole grains, which promotes healthy bowel function, reduces transit time, dilutes potential carcinogens [150], and serves as a crucial substrate for beneficial gut bacteria [151]. Reduced consumption of red and processed meats is also vital, as high intake is associated with increased CRC risk, possibly due to the formation of carcinogenic compounds during cooking and digestion [152,153]. Regular physical activity has multiple beneficial effects, including reducing inflammation, improving insulin sensitivity, and promoting healthy gut motility, all contributing to lower CRC risk [154]. Maintaining a healthy weight is crucial, as obesity is a significant risk factor for CRC [155], and limiting alcohol consumption and avoiding smoking are also linked to reduced risk [156]. Some studies suggest a protective role for adequate calcium and vitamin D intake, although the evidence is not conclusive for all populations [157], and the consumption of probiotics/prebiotics may also help by restoring healthy gut bacteria [158]. Tailoring these recommendations to different age groups is important; older adults may need to focus on maintaining adequate protein intake alongside fiber to combat age-related muscle loss (sarcopenia), while younger adults may benefit from early education on the dangers of processed foods and sedentary lifestyles.

Beyond lifestyle modifications, a more radical approach involves directly targeting the biological processes of colon aging to prevent or delay CRC onset. This is a rapidly developing field with potential interventions, such as senolytics (drugs that selectively eliminate senescent cells, which contribute to inflammaging and tissue dysfunction) [159]. Microbiome modulation, through strategies like prebiotics, probiotics, or fecal microbiota transplantation (FMT), may restore a more youthful and resilient gut ecosystem [160,161]. Targeting specific inflammatory pathways implicated in colon aging and CRC development, such as through the use of anti-inflammatory agents like aspirin, could also offer preventative benefits [162]. Research into the role of mTOR inhibitors in extending lifespan and reducing age-related disease, as well as therapies targeting circadian rhythm (clock-modulating therapies also show promise [163]. While these interventions are largely in the experimental stage, the potential for developing therapies that directly target colon aging is significant, particularly for individuals with genetic predispositions or other high-risk factors.

These research findings should also inform public health policies and recommendations to promote healthy aging and reduce the overall CRC burden. This includes promoting healthy lifestyle choices through public health campaigns that emphasize the importance of diet, exercise, weight management, and avoiding smoking and excessive alcohol consumption throughout the lifespan. Improving screening uptake, particularly in underserved populations, is crucial [164], and this includes addressing barriers to screening, such as cost, access to care, and lack of awareness. Public health guidelines should reflect the latest research on optimal screening ages and intervals, taking into account individual risk factors and life expectancy [165,166]. Finally, continued investment in research on colon aging and CRC prevention is essential to develop more effective and targeted interventions. Preventative interventions and strategies to promote healthy colon aging are of paramount importance for older adults. As the colon ages, the risk of CRC increases dramatically. Maintaining a healthy lifestyle, participating in appropriate screening, and potentially utilizing future interventions that target colon aging processes can significantly reduce this risk and promote overall health and well-being in later life.

## 3. Conclusions and Perspectives

### 3.1. Deepening Mechanistic Understanding of Colon Aging and CRC Interaction

A critical frontier in colorectal cancer (CRC) research lies in deepening our mechanistic understanding of how the aging process within the colon fuels carcinogenesis. While it is established that aging is a major risk factor, the precise molecular and cellular events that link age-related changes to increased CRC susceptibility remain incompletely understood. The hallmarks of aging, including genomic instability, telomere attrition, epigenetic alterations, loss of protein homeostasis, deregulated nutrient sensing, mitochondrial dysfunction, cellular senescence, stem cell exhaustion, and altered intercellular communication, are undoubtedly intertwined, but unraveling their specific interactions *within the unique context of the colonic microenvironment* is paramount [14]. Future studies should focus on how age-related changes in one hallmark influence the other, and promote tumor formation.

A crucial area of investigation is how aging disrupts the delicate balance of tissue homeostasis within the colonic epithelium. Beyond understanding age-related changes in intestinal stem cell function, immune surveillance, and the extracellular matrix (ECM), we need to determine how aging impacts the molecular mechanisms that maintain colonic cell identity and prevent lineage infidelity. What are the key tissue-specific transcription factors that safeguard the colonic phenotype, and how is their function or expression (potentially via epigenetic silencing like age-related DNA methylation) altered with age? Could the loss of such “identity-guarding” factors contribute to metaplastic changes, representing a potential pathway to malignancy? Furthermore, the interplay between age-related metabolic reprogramming within epithelial cells and these shifts in cell fate warrants exploration. The colonic microenvironment is a complex ecosystem, and understanding the specific roles and communication networks of each cell type—epithelial cells, fibroblasts, immune cells, endothelial cells, neurons, and the diverse microbial community—remains vital, particularly considering the distinct biological properties of the proximal versus distal colon. How these interactions change with age, potentially differing regionally, and promote or suppress CRC development needs further definition. This regional heterogeneity is critical, as the proximal and distal colon exhibit distinct embryological origins, luminal environments (e.g., microbial populations, bile acid concentrations), baseline immune tones, and metabolic functions, all of which can be differentially affected by the aging process. For instance, age-related epigenetic drift, DNA methylation patterns, and the accumulation of somatic mutations may vary along the colonic axis. Recent multi-omics studies in non-human primates by Wang et al. [12] have provided compelling evidence for such segment-specific aging, identifying distinct molecular signatures, including alterations in tryptophan metabolism (kynurenine and serotonin pathways), that differ between the aging proximal and distal colon and have functional implications for regional disease susceptibility. Understanding these differences is paramount, as they likely contribute to the well-documented heterogeneity in CRC, where proximal and distal tumors often exhibit different genetic (e.g., MSI, BRAF mutations more common proximally) and epigenetic (e.g., CIMP status) profiles, and can respond differently to therapies. Such regional aging insights could eventually inform more precise risk stratification models and perhaps even guide tailored screening approaches, for example, by highlighting the need for meticulous examination of specific colonic segments in individuals with certain aging biomarkers, or by aiding in the development of region-specific preventative strategies. The role of senescent fibroblasts, for example, and their SASP, requires more investigation [167,168,169].

Furthermore, most current research provides cross-sectional snapshots of the aging colon. Longitudinal studies, tracking changes in the colonic microenvironment and the microbiome over extended periods in both humans and animal models, are essential. This temporal perspective is crucial for identifying the *early* molecular and cellular events that precede and drive CRC development, potentially revealing critical windows for intervention.

Finally, while the association between gut dysbiosis, aging, and CRC is well-established, the specific microbial players and mechanisms involved require further elucidation. Research must move beyond simply identifying associations and delve into the *causal* relationships. How does the aging gut microbiome differ functionally from a younger microbiome in promoting CRC? Are there specific “gerobugs” or “oncomicrobes” that are particularly influential in the context of age-related CRC? Detailed mechanistic studies, potentially employing gnotobiotic animal models and advanced in vitro systems that mimic the colonic microenvironment, are needed to address these questions, and characterize the microbiome differences between younger and older onset CRC [170,171].

### 3.2. Tools of Discovery in Colonic Aging and Colorectal Cancer: Single-Cell Omics and Organoid Models

Single-cell sequencing technologies, including single-cell RNA sequencing (scRNA-seq), single-cell ATAC sequencing (scATAC-seq), and spatial transcriptomics, are revolutionizing our understanding of cellular heterogeneity and dynamic processes in colon aging and CRC [172]. Traditional bulk sequencing methods provide an average signal from heterogeneous cell populations, masking crucial cell-type-specific alterations and the complex interplay between different cell types. By contrast, single-cell approaches can precisely deconvolute the cellular landscape of the aging colon, delineating how individual cell types—such as intestinal stem cells (ISCs), various differentiated epithelial cells (enterocytes, goblet cells, enteroendocrine cells), diverse immune subsets (T-cells, B-cells, macrophages, dendritic cells, myeloid-derived suppressor cells), and stromal fibroblasts—respond to aging at a molecular level. This enables the identification of rare cell populations that may be particularly susceptible to age-related dysfunction or oncogenic transformation, which would be undetectable in bulk samples. Furthermore, scATAC-seq can reveal cell-type-specific epigenetic landscape changes, providing insights into how chromatin accessibility and gene regulation are altered during aging and CRC development in a resolution previously impossible. Spatial transcriptomics adds another crucial dimension by preserving the tissue architecture, allowing researchers to understand where these molecular and cellular changes occur within the colonic crypts and microenvironment, revealing localized aging niches or nascent tumor foci [173]. These high-resolution analyses are critical for identifying subtle shifts in cellular identities, aberrant differentiation trajectories (ISC lineage infidelity), altered cell–cell communication networks (via ligand–receptor interaction analysis), and precise pro-inflammatory or pro-tumorigenic signatures within specific cellular compartments in the aging colon, ultimately leading to the discovery of novel, cell-type-specific biomarkers and more targeted therapeutic strategies.

Complementing single-cell analyses, sophisticated organoid models, particularly patient-derived organoids (PDOs), offer unparalleled opportunities to study colon aging and CRC in vitro in a physiologically relevant 3D context [174]. Unlike traditional 2D cell cultures, organoids recapitulate the complex cellular architecture, stem cell hierarchy, cell–cell interactions, and functional properties of the native colon epithelium. This allows for the faithful modeling of age-related alterations: organoids derived from aged human donors exhibit hallmarks of aging (reduced stem cell function, altered differentiation patterns, increased senescence markers), and conversely, aging processes can be accelerated or reversed in vitro in younger organoids using specific genetic manipulations or pharmacological interventions [175]. For CRC research, PDOs derived directly from tumors retain the genetic and phenotypic heterogeneity of the original tumor, enabling personalized drug screening and the identification of age- and sex-specific vulnerabilities to treatments. Moreover, the modular nature of organoids allows for the creation of complex co-culture systems that incorporate key components of the colonic microenvironment, such as immune cells (to study inflammaging and immune surveillance), fibroblasts (to investigate ECM remodeling and SASP), and even specific microbial communities (to model dysbiosis and its impact on epithelial aging and transformation) [176]. These systems provide a controlled, manipulable platform to dissect the causal relationships between specific aging hallmarks, microenvironmental factors, and CRC initiation/progression, while also facilitating the high-throughput testing of preventative and therapeutic agents.

The synergy between single-cell multi-omics and organoid models creates a powerful research paradigm. By identifying specific cellular and molecular changes in the aging colon using single-cell resolution, researchers can then functionally validate these findings in organoid models, testing causal links and evaluating potential interventions in a human-relevant system. This integrated approach will accelerate the discovery of novel biomarkers for early detection and risk stratification, unravel the intricate mechanisms by which colon aging drives CRC, and ultimately inform the development of highly targeted and effective personalized preventative and therapeutic strategies for colorectal cancer.

### 3.3. Translating Colon Aging Insights into Clinical Applications

The expanding knowledge of colon aging and its intricate relationship with colorectal cancer (CRC) holds immense promise for improving clinical practice, particularly in the areas of early detection, risk stratification, and prevention (Figure 5). However, translating these research findings into tangible clinical benefits requires concerted effort and further investigation.

A primary focus is the validation and implementation of novel biomarkers. Numerous studies have identified potential molecular-, cellular-, and microbiome-based markers associated with colon aging and increased CRC risk. These include DNA methylation patterns (*SDC2* methylation in stool samples), circulating cell-free nucleosomes with specific epigenetic modifications, ctDNA methylation haplotype patterns (ColonES [137]), age-related differentially expressed genes (*DLX2* and *PCOLCE2* [177]), aberrant miRNA expression [139], and specific fecal microbial signatures or metabolites (*p-cresol* and *3(4H)-DBZ* [141]). While promising, these biomarkers require rigorous validation in large, diverse, and well-defined populations before they can be incorporated into routine clinical practice. This includes determining their sensitivity, specificity, and positive and negative predictive values across various age groups, ethnicities, and risk profiles. Further complicating this is the Centers for Medicare and Medicaid Services (CMS) stringent requirements for coverage of blood based biomarker tests [178].

Another critical step is the integration of colon aging biomarkers into existing CRC risk prediction models. Current models predominantly rely on traditional risk factors such as age, family history, and lifestyle factors. Adding measures of biological age, derived from molecular or cellular features of the colon, could significantly improve the accuracy of these models (Figure 5). This would enable more personalized risk stratification, allowing for tailored screening recommendations. Individuals with “accelerated” colon aging, regardless of their chronological age, could be identified as candidates for earlier or more frequent screening, while those with “younger” colons might be spared unnecessary procedures.

Despite significant advancements in the discovery of aging biomarkers, their widespread clinical translation for CRC risk assessment and screening remains constrained by several critical limitations. Firstly, there is a lack of robust validation and standardization, as many promising biomarkers, such as DNA methylation clocks, inflammatory markers, and gut microbiota features, have primarily been identified in small, homogeneous cohorts. Their generalizability, sensitivity, specificity, and predictive value are often not yet sufficiently validated in large, diverse populations spanning different ethnicities, geographies, lifestyles, and comorbidities, hindering cross-platform comparability and clinical reliability. Secondly, the clinical relevance and incremental benefit of these biomarkers are frequently unclear; while they reflect biological aging, their added value over established clinical risk factors (e.g., chronological age, family history, polyp history) in guiding specific clinical decisions—such as optimal screening initiation, interval adjustments, or the necessity for invasive procedures—requires stronger evidence. Thirdly, the technical complexity and high cost associated with advanced multi-omics-based detection methods (e.g., whole-genome methylation, single-cell sequencing) severely limit their accessibility and widespread adoption in routine clinical settings, particularly in resource-constrained regions. Overcoming these limitations is paramount for harnessing the full potential of aging biomarkers in improving CRC prevention, early detection, and personalized management.

Research on colon aging can inform the optimization of screening strategies. Existing guidelines largely rely on chronological age for recommending screening initiation and intervals. However, incorporating measures of biological age and individual risk factors could lead to more efficient and cost-effective screening protocols. For example, some data might support later screening cutoff ages [143], or variable screening frequency based on FIT results [179].

The development and implementation of such personalized strategies will depend on not only biomarker validation but also on addressing practical considerations like colonoscopy capacity and regional variations in healthcare resources.

The development of non-invasive screening methods, such as liquid biopsies and improved stool-based tests [180,181] are particularly important, especially for older adults who may be less tolerant of invasive procedures (Figure 5). These methods could increase screening participation and improve early detection rates in this high-risk population.

Finally, translating research on colon aging and CRC into clinical practice requires a commitment to conducting rigorous intervention trials. Promising preclinical findings, such as the potential of senolytics or microbiome modulation to prevent CRC, need to be tested in well-designed human studies. These two key aging-related pathways are actively being explored in clinical studies, utilizing specific biomarkers to track their impact and the efficacy of interventions:

Cellular Senescence: The accumulation of senescent cells (often called ‘zombie cells’), which secrete harmful inflammatory factors (SASP), is linked to aging and various age-related diseases. Clinical studies are testing senolytic drugs (Dasatinib + Quercetin, Fisetin) to selectively eliminate these cells. Biomarkers like plasma IL-6, TNF-alpha, and GDF15 (components of SASP) are measured to monitor the effectiveness of these treatments and the systemic senescent burden in trials for conditions like idiopathic pulmonary fibrosis, osteoarthritis, and Alzheimer’s disease [182].

Mitochondrial Dysfunction and NAD+ Metabolism: Declining mitochondrial function and reduced levels of NAD+ (a vital coenzyme for energy metabolism) are central to aging. While direct NAD+/NADH ratios are complex to measure in humans, NAD+ precursors like Nicotinamide Riboside (NR) [183] and Nicotinamide Mononucleotide (NMN) [184] are being widely tested in clinical trials. The focus is on their impact on various physiological outcomes, including improvements in muscle function, metabolic health (insulin sensitivity), and cognitive performance, serving as practical proxies for enhanced mitochondrial health. Furthermore, a growing number of natural compounds and naturally derived pharmaceuticals are demonstrating significant clinical potential. Notable examples encompass metformin, glucagon-like peptide-1 receptor agonists (GLP-1RAs), TORC1 inhibitors (rapamycin and its analogs), and spermidine [185].

In summary, the ultimate goal is for research on colon aging to move beyond simply understanding the mechanisms of disease to actively improving CRC, enabling the development of personalized, effective, and age-appropriate strategies for early detection, risk stratification, and prevention.
